# On the emergence of antibacterial and antiviral copper cold spray coatings

**DOI:** 10.1186/s13036-021-00256-7

**Published:** 2021-02-24

**Authors:** Bryer C. Sousa, Christopher J. Massar, Matthew A. Gleason, Danielle L. Cote

**Affiliations:** Materials Science and Engineering Program, Department of Mechanical Engineering, Worcester Polytechnic Institute, 100 Institute Road, Worcester, MA 01609-2280 USA

**Keywords:** Copper, Cold spray, Antimicrobial surfaces, Biocidal contact killing, Viricidal contact inactivation, Atomic ion diffusion pathways, Antipathogenic mechanisms, Microstructures, Biomaterials

## Abstract

In this literature review, the antipathogenic properties and contact-mediated antibacterial and antiviral performance of copper cold spray surfaces are assessed and compared with alternative antimicrobial materials that are able to kill and/or inactivate infectious agents via direct contact. Discussion is also provided concerning the suitability of copper cold spray material consolidations as biocidal and viricidal surfaces that retain long-term functionality as a preventative measure against fomite transmission of pathogenic agents and hospital-acquired infections from contaminated high-touch surfaces. Numerable alternative antimicrobial coatings and surfaces that do not rely upon the oligodynamic action of copper are detailed. Given the ongoing need for recognition of said alternative antimicrobial materials by authoritative agencies, such as the U.S. Environmental Protection Agency, the relevant literature on non-copper-based antipathogenic coatings and surfaces are then described. Furthermore, a wide-ranging take on antipathogenic copper cold spray coatings are provided and consideration is given to the distinctive grain-boundary mediated copper ion diffusion pathways found in optimizable, highly deformed, copper cold spray material consolidations that enable pathogen inactivation on surfaces from direct contact. To conclude this literature review, analysis of how copper cold spray coatings can be employed as a preventative measure against COVID-19 was also presented in light of on-going debates surrounding SARS-CoV-2’s non-primary, but non-negligible, secondary transmission pathway, and also presented in conjunction with the inevitability that future pathogens, which will be responsible for forthcoming global pandemics, may spread even more readily via fomite pathways too.

## Introduction and background

Some have argued that the basic ideas underpinning cold gas-dynamic spray (cold spray) pre-date the conceptualization, research, and formalization that was performed by Papyrin et al. in the late twentieth century (given the early twentieth century documentation provided as part of two U.S. patent applications [1, 2]); however, cold spray was initially developed in its modern form at the Institute of Theoretical and Applied Mechanics of the Russian Academy of Sciences in Novosibirsk during the mid-1980s and early 1990s [3]. Investigations into applying cold spray as a rapid consolidation and coating technology was adopted shortly after Papyrin et al.’s syncretistic discovery was reported upon, which ultimately led to the eventual progression towards component restoration and structural repair applications that dominates the contemporary commercial and defense landscape associated with cold spray deposition. Generally speaking, cold spray is a solid-state materials consolidation technology that utilizes particulate feedstocks, which are transported using a heated carrier gas stream until departing a convergent-divergent de Laval nozzle and supersonically impacting a substrate. As supersonically impacted particulates bond to the substrate, particle-to-particle bonding occurs as the process continues to consolidate powder in a layer-by-layer fashion.

**Fig. 1:**
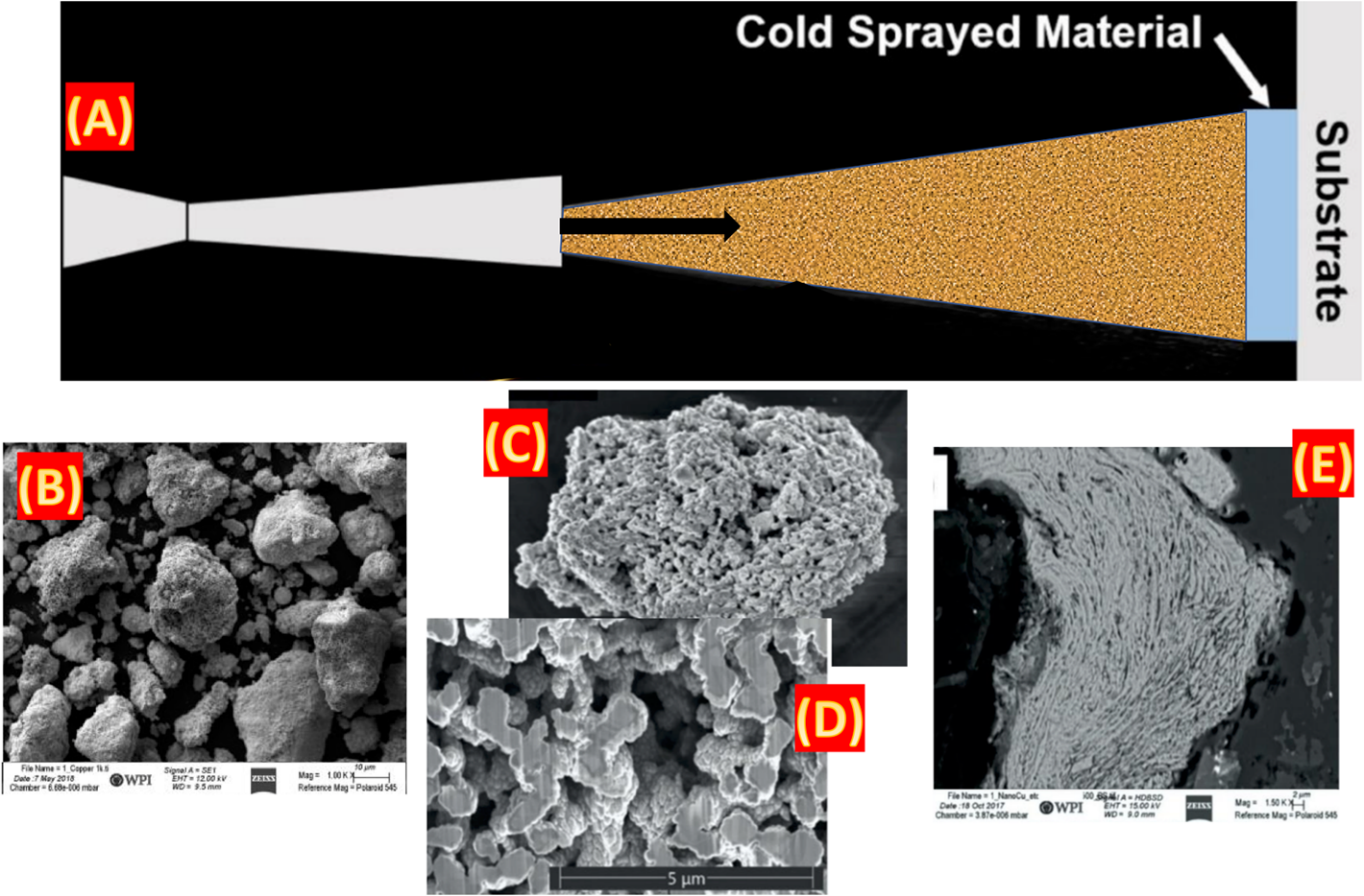
Schematic of the copper cold spray process (**a**). The nanostructured spray-dried agglomerated copper feedstock utilized to produce an antimicrobial cold spray coating is shown in (**b**), while (**c**) presents a single particulate, **d** presents a cross-section of a single particle, and (**e**) is a cross-section of the resultant coating

Cold spray processing parameters vary, from the type of material used to construct the nozzle to the selection of the powder composition and the gas source. Normally, inert gases are used, such as helium or nitrogen. Feedstock particulate systems for cold spray typically maintain a diameter range of approximately 10 μm to 100 μm. Particles reach velocities between 300 m/s and 1200 m/s. A wide range of materials may be cold sprayed, which include composites, copper, aluminum, steel, and titanium, and are selected according to the requirements of a given application. There are a significant number of properties that can influence the high strain rate impact induced and deformation-driven bonding, ranging from the feedstock morphology and powder size distribution to carrier gas type, temperature and pressure, as well as deposition efficiency, applicator design, and nozzle type. These are further compounded by traditional manufacturing factors like traverse speed, step size, etc. Keeping the aforementioned in mind, Fig. 1 schematically depicts a simplified version of the cold spray manufacturing process itself to more clearly capture the material’s perspective for copper cold spray from spray-dried powder to the resultant consolidated coating as an example.

As an antimicrobial coating application, cold spray can be deposited onto various substrate materials at various processing temperatures and pressures, thus allowing for exceptionally high versatility in applications. Additionally, because cold spray can be deposited through computer-generated pathways mounted on a robotic arm or as a handheld applicator, generating highly antimicrobial, or functional surfaces on preexisting parts is a highly feasible opportunity for improvement to both new and preexisting parts regardless of geometry. Reemergence of copper as an antimicrobial touch surface application was investigated by Dick et al. at Battelle Columbus Laboratories [4]. Since then, the capitalization of materials with antimicrobial properties has been further explored breaking away from strictly metallic systems to polymeric and inorganic materials. While it has been demonstrated that zinc and silver-containing systems have antimicrobial properties, copper has demonstrated significantly higher kill rates in a broader variety of bacteria and deactivation in viruses. Moreover, copper-containing implants have shown to be nontoxic in small concentrations [5], suggesting that antipathogenic copper-based biocompatible surfaces can be useful outside of fomite-mediated pathogen transmission prevention too.

### Antipathogenic copper cold spray surfaces

**Fig. 2:**
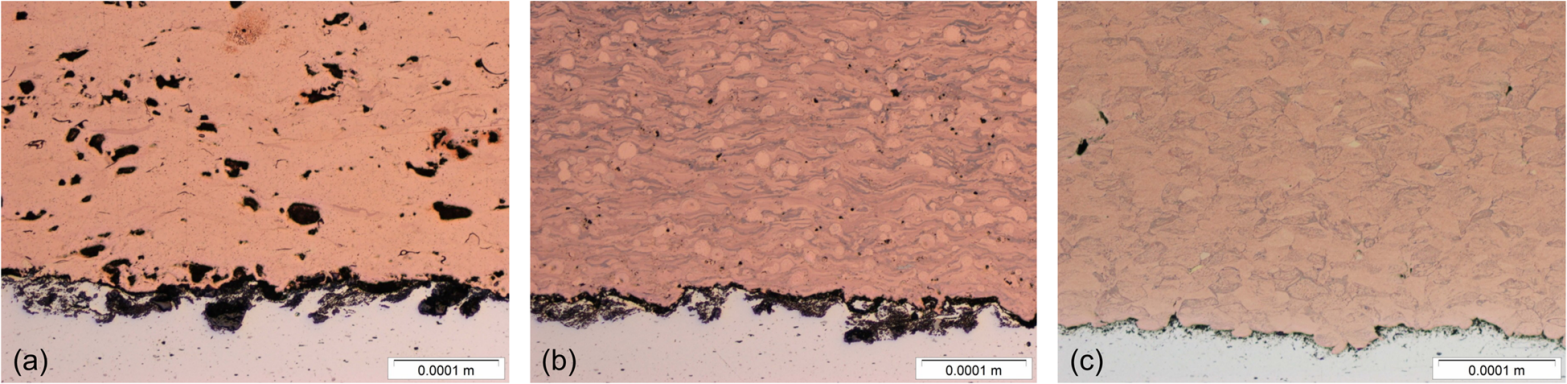
Resultant microstructures of each of the coatings procured and was adopted from the open source publication cited herein as reference [6]

From an experimentally minded vantage point, Champagne et al. evaluated the antimicrobial capabilities and properties of three uniquely procured copper coatings. Each of the three aforementioned copper coated surfaces were manufactured by way of three thermal spray-based material processing techniques [6]. More specifically, Champagne et al. considered plasma spray processed copper coated surfaces, arc spray processed copper coated surfaces, and finally cold spray materials consolidation processed copper coated surfaces as part of Champagne et al.’s original research concerning antipathogenic copper coatings. Depositing each of the three coatings until a thickness of approximately 1 mm was reached, the plasma sprayed, arc sprayed, and cold sprayed coatings were applied to an aluminum alloy, which was comparable with aluminum-based material systems typically affiliated with hospital and/or medical settings. Beyond the fact that Champagne et al. was motivated to study various antimicrobial copper coatings produced via thermal spray techniques in general, Champagne et al. also wanted to test their postulation that the distinctive microstructures affiliated with each of the coatings would attain different antimicrobial efficacies based upon each of the three materials unique microstructures. Upon surveying the microstructures associated with each respective copper coated surfaces, Champagne et al. noticed that “[differences] in microstructure are clearly evident, suggesting that differences in biological activity may also occur” [6]; thus, in line with Champagne et al.’s postulated hypothesis. Figure 2 presents the resultant microstructures of each of the coatings procured by Champagne et al. and was adopted from the open source publication cited herein as reference [6].

Once the plasma sprayed, arc sprayed, and cold sprayed copper coatings were structurally inspected via microscopy-based methods following successful deposition, Methicillin-resistant *Staphylococcus aureus* (MRSA), was selected as the infectious agent of interest for proof-of-concept exploration. Antimicrobial efficacy evaluation was pursued by way of inoculating MRSA on the three copper coated and thermally sprayed surfaces. Thereafter, the inoculated specimens were held for 2 h at room temperature and under standard ambient conditions. Then, the bacterial cells that survived the direct exposure to the copper coated surfaces were cultured in a manner consistent with the EPA protocol of relevance known as “Test Method for Efficacy of Copper Alloy Surfaces as a Sanitizer.” Ultimately, the resultant percent of surviving MRSA after 2 h of exposure time to the three thermal spray copper coatings was consistent with their assertation that differences in biological activity would follow from the differences in microstructures associated with each thermally sprayed surface. While the plasma sprayed surface killed less than 90% of the exposed MRSA after 2 h, the cold sprayed copper killed more than 99.999% of the bacterial agent after the same duration of time (and potentially 100% of the inoculated MRSA since the testing limitation was surpassed).

**Fig. 3:**
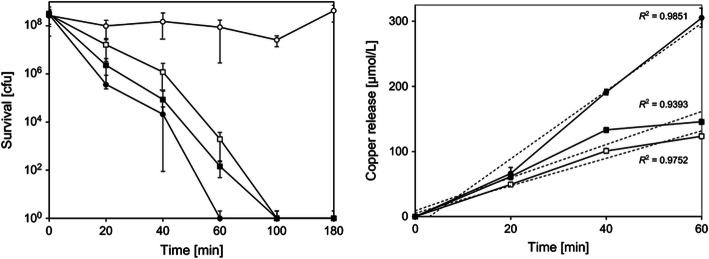
Copper release rate as a function of time, as well as the survival of *E. coli* (strain K12) inoculated upon various copper surfaces with observable differences in surface roughness and topographies [12]

Since this review will retouch upon the work of Champagne et al. at a later point, it behooves us to note that Rutkowska-Gorczyca studied the microstructure of a biocidal copper and titanium-dioxide composite surface built using low pressure cold spray [7]. Before Rutkowska-Gorczyca published [8], Sanpo et al. assessed a copper and zinc-oxide composite cold spray surface to facilitate microbial contact killing and the proscription of *Cobetia marina* binding to the available surfaces submerged as part of the maritime ships of interest [9]. Outside of Sanpo et al.’s research, continued accumulation of data attests to the fact that direct surface contact between a microbe and contact killing/inactivating copper surface plays a substantial part in capturing optimal antipathogenic efficacy [10]. Such substantiating data follows from scholarly demonstrations of the fact that surface topography also, tends to impact the biocidal, and in some cases viricidal, activities of a given antimicrobial surface [11]. Accordingly, Fig. 3 presents the copper release rate as a function of time, as well as the survival of *Escherichia coli* (strain K12) inoculated upon various copper surfaces with observable differences in surface roughness [12].

Concomitantly, surface chemistry, surface physics and surface energetics have also been identified as contributing factors in so far as intimate and direct pathogen-copper contact mediated killing and/or inactivation is concerned. In fact, Vucko et al. exhibited the antifouling activity of a high-density polyethylene that was “metallized” using copper particulates as well as the cold spray materials consolidation process [13]. Vucko et al. found that after 250 days of continuous field-testing, the high-density polyethylene, which was metallized with copper powder as an embedded constituent in the coating, killed or inactivated all of the hard fouling agents. From Vucko et al.’s experimental research, one may more readily appreciate how the introduction of an oligodynamic metal, such as copper, prevents sustained adherence of microorganisms to a metallized high-density polyethylene-copper powder composite surface. Also remarkable is the fact that while the bacterial agent was attempting to adhere to the metallized plastic-copper composite surface, the proliferation of the bacteria that made it to the antibacterial coatings was inhibited through direct contact even when surrounded by an otherwise oceanic environment. Though Vucko et al. have brought the focus of this section of the review closer to the foci of antimicrobial, commercially pure, copper cold spray coatings, like those associated with Champagne et al.’s publications, additional work by El- Eskandrany et al. warrants consideration as another non-pure copper antibacterial cold spray approach. Said otherwise, from a point of view that was relevant to the healthcare and agricultural industries, El-Eskandrany et al. explored the use of a copper-containing (Cu_50_Ti_20_Ni_30_) alloyed metallic glass powder for antibacterial cold spray applications [14]. The reader is encouraged to explore such work after finishing the present review article for adequate appreciation of El- Eskandrany et al.’s novel approach.

**Fig. 4:**
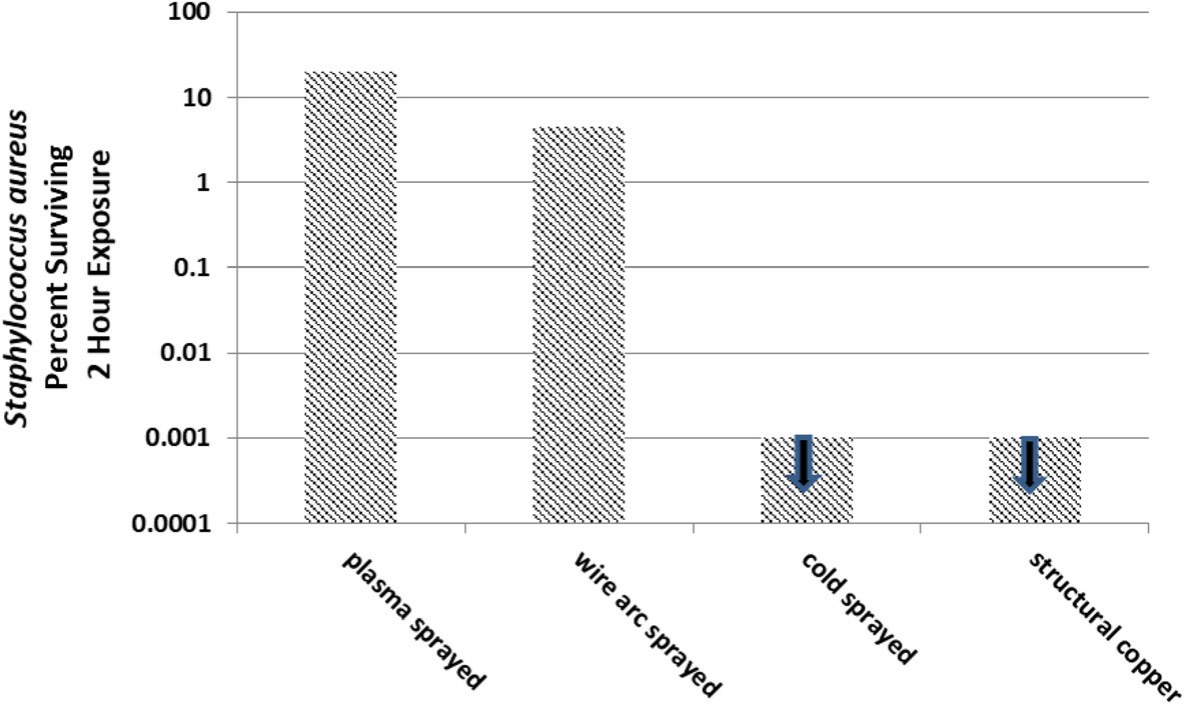
Adopted from [16] to highlight “Percent… MRSA… surviving after a two-hour exposure to copper surfaces”

In any case, after Champagne et al. published their 2013 proof-of-concept study, the authors connected with Sundberg et al. in 2015 to study the capability of commercially pure copper cold spray surfaces to inactivate influenza A virus [15]. The collaborative effort attempted to showcase copper cold spray coatings’ ability to thwart surface-contact fomite transmission of a viral pathogen. Influenza A was reportedly chosen since surface-contact fomite transmission may ensue 72 h after a non-antiviral surface is exposed to the virus. Sundberg et al. went a step further by way of also varying the copper powder fed into the high-pressure cold spray system to realize commercially pure copper cold spray coatings, which in one case contained the same microstructure ascertained in 2013, whilst a second copper cold spray coating possessed a nanostructured and agglomerated crystallinity. While Fig. 2(c) presents a cross-section of the original copper cold spray coating’s microstructure, Fig. 1(e) captures the nanostructured coating’s cross-sectional microstructure after chemical etching through the use of a scanning electron microscope. Intriguingly, the nanostructured copper cold spray coating achieved a 99.3% decrease of infectious influenza A virions after 2 h of exposure (according to the aforementioned EPA protocol of relevance). In contrast with the nanostructured cold spray coating, the ultra-fine/fine-grained copper cold spray surface associated with the earlier MRSA study was found to accomplish a 97.7% reduction of infectious influenza A pathogens, thus attesting to the fact that Champagne et al.’s 2013 hypothesis that antimicrobial performance is a function of microstructure also holds for viral pathogens too. Figure 4 presents the results from Champagne et al.’s original 2013 study as well.

In 2019, Sundberg offered initial characterization and research that identified several material properties as being influential upon the antimicrobial efficacy of pure copper cold spray surfaces in a doctoral dissertation, which was entitled “Application of Materials Characterization, Efficacy Testing, and Modelling Methods on Copper Cold Spray Coatings for Optimized Antimicrobial Properties,” and publicly defended at Worcester Polytechnic Institute [8]. Nevertheless, Champagne et al. sustained their examination of copper cold spray surface contact killing and/or inactivation efficacies by summarizing their 2013 to 2019 findings for the purpose of substantiating their second hypothesis that the dislocation density created by cold spray consolidation of copper powder is the microstructural feature liable for cold spray’s superior antipathogenic efficacies relative to other manufacturing methods [16]. Said otherwise, Champagne et al. conveyed dislocation density as the microstructural constituent most responsible for copper cold spray coatings’ antibacterial and antiviral behaviors. However, a 2020 article by Sousa et al. gave an alternate microstructure-mediated mechanistic framework which classified the surface area concentration of polycrystalline grain boundaries of the consolidated coated material as being more telling of the surfaces’ antimicrobial behavior than dislocation density alone [17].

Rather than disregarding dislocation density when formulating a mathematical relation for the effective copper ion diffusivity, wherein the copper microstructure with the greatest effective copper ion diffusivity corresponds with the most antipathogenic crystal structure, Sousa et al. discovered the fact “that the grain boundary contribution to the effective diffusivity of copper ions in the nanostructured material outperforms the diffusivity of dislocation pipe diffusion by an order of magnitude” [17], where Champagne et al. presented the effective copper ion diffusivity as,

$$ {D}_{eff}={D}_o\left[1+\pi {a}^2{\rho}_d\left(\frac{D_d}{D_o}-1\right)\right] $$

such that *D*_*eff*_ is the effective ionic diffusivity, *D*_*o*_ is the lattice diffusivity, *D*_*d*_ is the pipe dislocation diffusivity, *ρ*_*d*_ is the dislocation density, and *a* is the average dislocation radius. On the other hand, Sousa et al. incorporated and demonstrated essential refinements to the way in which an effective ionic diffusivity need be presented to represent the microstructures associated with the copper cold spray coatings such that,

$$ {D}_{eff}={f}_i{D}_i+{D}_l+\frac{\delta_{gb}\left(w-d\right)}{wd}{D}_{gb}+\rho a{D}_c $$

where *δ*_*gb*_ is the grain boundary thickness, *d* is the average grain or sub-grain size, *ρ* is the dislocation density, *w* is the line width, *a* is the cross-sectional area of the dislocation cores associated with pipe diffusion, *f*_*i*_ is the fraction of atoms undergoing atomic diffusion via the additional interfacial pathways within a material, *D*_*i*_ is the diffusion coefficient for atomic diffusion through additional interfaces within a material, *D*_*l*_ is the diffusion coefficient associated with atomic diffusion through the lattice, *D*_*gb*_ is the diffusion coefficient for atomic diffusion through the grain boundaries, and *D*_*c*_ is the diffusion coefficient for atomic diffusion through dislocation cores, also known as “pipe diffusion,” as readily pointed out by Champagne et al. too.

**Fig. 5:**
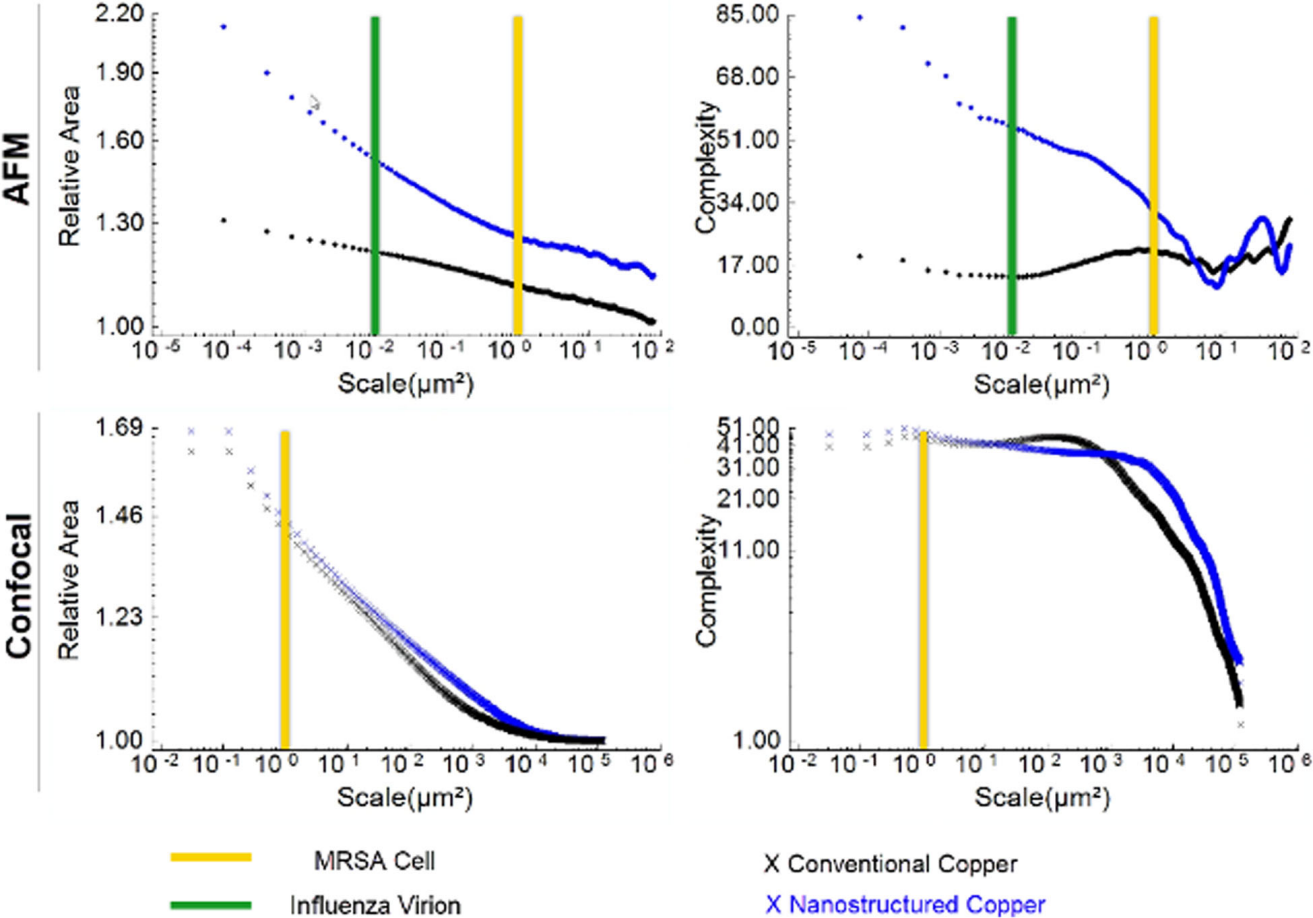
Multiscale area and complexity characterizations (ASME B46.1, ISO 25178-2) of topographies measured with AFM and confocal microscopy. MRSA (bacteria) and influenza A (virus) sizes given for size-scale reference [17]

Outside the realm of strictly microstructure-mediated contact killing and inactivation mechanisms, in 2019, Sundberg et al. considered the microscale and nanoscale roughness of the ultra-fine/fine-grained copper cold spray surfaces as well as the nanostructured copper cold spray surface manufactured using spray-dried powder as the feedstock material [18]. Sundberg et al. performed said characterization via atomic force microscopy and three-dimensional confocal microscopy in an attempt to more clearly, coherently and readily appreciate the enhanced viricidal activity of the nanostructured copper cold spray coating vs. alternative copper-based viricidal surfaces and the ultrafine/fine-grained copper cold spray surface. Also, in 2019, Sundberg et al. supplemented their prior work with copper ion release rate analysis and copper cold spray coating corrosion evaluation. Said corrosion studies were pursued in an attempt to better appreciate the chemical reactivity at the surface [19]. Finally, Fig. 5 presents the resultant relative area vs. scale and complexity vs. scale plots for the two copper cold spray coatings of relevance, as reported by Sousa et al., and compared against the size of an average MRSA cell and an average influenza capsid.

As already alluded to herein, surface chemistry and surface oxide species are also known to affect the role of copper contact killing/inactivation in general as well as copper cold spray surface antimicrobial efficacy in particular. In so far as the copper-oxide species present at the surface are concerned, which may take the chemical form of Cu_2_O and CuO, each individual oxide species effects the biocidal and viricidal capacity of a pure copper material in a variety of ways. Both Cu_2_O and CuO have previously been found to diffuse copper ions at different rates [20]. Cu_2_O and CuO have also been found to control the copper ion species released from the oxide or diffused through the copper oxide, where Cu^1+^ or Cu^2+^ is released more or less favorably depending upon the copper-oxide species present. This is of notable significance since Cu^1+^ ions are hypothesized as maintaining greater antipathogenic tendencies than Cu^2+^. Returning to the matter of copper-oxide species, CuO has previously been found to maintain a lower copper ion release rate in general than that of Cu_2_O. Given the apparent importance of copper-oxide surface species and copper ion speciation research was also performed by Sundberg et al. to inspect the copper cold spray coatings in greater detail as well [21]. More to the point, using X-ray photoelectron spectroscopy, some of the authors of this review characterized the surface oxide species located upon the conventional and nanostructured antimicrobial copper cold sprayed surfaces. As a result, Cu^2+^ was found to comprise the spectra related to the ultrafine/fine-grained copper cold spray coating, whereas Cu^1+^ was found to comprise the spectra for the nanostructured copper cold spray coating, which is in line with the amplified antimicrobial capacity accompanying the nanostructured copper cold spray surfaces compared to the conventional copper cold spray surface.

### Alternative antipathogenic cold spray coatings

While copper is obviously the antimicrobial metal of relevance and relevance herein, one may also note that antipathogenic surfaces have previously been presented within the literature that used non-copper-based cold spray feedstock materials too. For more details on said non-copper-based alternatives that also employed cold spray to generate bio-functional and antimicrobial surfaces, one ought to consider the subsection of Vilardell et al.’s 2015 review article [22], titled “Antibacterial/antimicrobial coatings.” In any case, while microscale and nanocrystalline copper cold spray has demonstrated the most promising antibiological properties, other material depositions including metallic and polymeric compounds have been investigated. In a study conducted by Tamai et al. P_2_O_5_-SiO_2_-Al_2_O_3_-CaO was investigated as an inorganic antimicrobial blended feedstock accompanied with 20 to 30% ZnO as the functional antimicrobial material [23]. Tamai et al.’s study investigated antimicrobial properties with relation to *S. aureus*, *Pseudomonas aeruginosa*, and *Klebsiella pneumoniae*, with suitably high efficacy in relation to the *S. aureus* and *P. aeruginosa* bacterium; however, the cold spray ZnO containing composite coating was less successful in so far as the bacterial agent *K. pneumoniae* is concerned. *S. aureus* and *P. aeruginosa* both experienced an approximately four orders of magnitude decrease in bacteria present after 24 h while *K. pneumoniae* only decreased roughly two orders of magnitude. The antimicrobial effect against *K. pneumoniae* was less than that against other species of bacteria. However, one of the unique properties of ZnO is that it exhibits photocatalytic properties meaning that this could be a mechanism which can promote antimicrobial properties in this coating.

While the application expands outside of the traditional scope of antimicrobial coatings in the sense of conventional high touch surfaces, polymeric membranes for biofunctionalization consisting of cellulose acetate, polyamide, and polyvinylidene-difluoride were cold sprayed as bio-fouling resistant coatings. When silver nanoparticles were impregnated into this membrane and sprayed, surface patterns generated a functionalized surface that reduced the permeation of microbes [24]. On the other hand, a chitosan-copper complex was tested by Sanpo et al. according to [25–27], investigated hydroxyapatite doped with nanophase silver, which was also sprayed demonstrating that with increasing hydroxyapatite-silver/PEEK nanopowder concentration the cold spray coating became increasing antimicrobial with pure glass having approximately 0.9E+ 09 *E. coli* compared to 1.0E+ 07 hydroxyapatite-silver/PEEK after 24 h. Noppakun then demonstrated exceptional Gram-positive antimicrobial properties when compared to Al-ZnO and ZnO. The copper addition to chitosan demonstrated a 70% reduction in *E. coli* demonstrating the feasibility of depositing the biopolymer with copper when blended with aluminum. In this work there was a seven order of magnitude drop in *E. coli*.

### Additional antipathogenic systems of interest

#### Non-copper-based alternative antimicrobial materials

The critical need for functionalized antipathogenic material solutions for use in biomedical and healthcare settings has continued to garner greater attention as the state of antibiotic resistance concurrently worsens [28]. To fully appreciate the scope and magnitude of the antibiotic resistance “nightmare scenario,” Ventola noted that “MRSA kills more Americans each year than HIV/AIDS, Parkinson’s disease, emphysema, and homicide combined,” [29], a stunning statistic that puts the antibiotic resistance crisis into its proper context. Such an immediate need for antimicrobial, antibacterial, antiviral, and in some cases antifungal, materials and coatings that are, in particular case scenarios, even more exotic than that of nanostructured copper cold spray coatings, for example, are not only due to the public health threats associated with complete antibiotic resistance. Rather, the incidence of highly transmissible, potentially life-threatening and contagious viral and bacterial outbreaks, like that of the present SARS-CoV-2 global pandemic, also motivates creative materials design, functionalization and applied research and development to be undertaken with respect to the creation and identification of prospective alternative antimicrobial surfaces. That being said, the unique alternative antipathogenic materials and coatings presented within the relevant scholarly literature are widespread, unapproved by regulatory agencies such as the EPA and CDC (for the most part) and achieve varying degrees of viability when cost, complexity, potential human toxicity and mechanical integrity are considered holistically. Nevertheless, even with the aforementioned in mind, noteworthy examples of the alternative antipathogenic materials identified within relatively recent academic literature are subsequently detailed.

Sikder et al. reported upon the successful creation of a trimagnesium phosphate hydrate nanofilm that was of a single phase and doped with silver for use as an antibacterial nanosheet coating [30], which was applied to poly(ether-ether-ketone), a common material used in biocompatible implants [30]. Sikder et al. demonstrated the enhanced synergy between biomechanical suitability of the implant material that follows from the microwave-irradiation mediated deposition of their nanosheet coatings to an approximate thickness of 650 nm and enhanced osteoblastic cell adhesion via in-vitro MC3T3 cell analysis. The silver-doped version of Sikder et al.’s trimagnesium phosphate hydrate nanosheet coating was then tested for antibacterial efficacy and antimicrobial performance using Gram-negative *E. coli* and Gram-positive *S. aureus*, which are bacterial agents considered to be common strains associated with hospital-acquired infection, whilst also confirming non-cytotoxicity through 3-(4,5-Dimethylthiazol-2-yl)-2,5-diphenyltetrazolium bromide assaying coupled with microscopy-based analysis. In conjunction with the quickly cited silver-doped nanosheets, notable antimicrobial, biocidal, and antiviral coatings, surfaces and materials have been considered as potential contact killing/inactivating bio-functional materials, which incorporate titanium dioxide and silver composite films, TiCaPCON films with embedded zinc, platinum and/or silver, NO_x_ emitting coatings, graphene nanoplatelets, cross-linked ionic polymer coatings, together with a number of other approaches.

Regarding the titanium dioxide nanoparticle and silver nanoparticle composite films, Li et al. volatized a solvent to fabricate polylactide titanium dioxide nanoparticle and blended polylactide titanium dioxide nanoparticle and silver nanoparticle composite antimicrobial packaging films [31]. Even though the blended composite films developed by Li et al. resulted in a less transparent film, the inclusion of titanium dioxide nanoparticles as well as silver nanoparticles enhanced the packaging films thermal stability according to thermogravimetric analysis. As for antibacterial capacity, acceptable antimicrobial ability was found to be associated with the blended polylactide titanium dioxide nanoparticle and silver nanoparticle films using *E. coli* and *Listeria monocytogenes* as the bacterial pathogens. While Li et al. found that their nano-blend composite films released nanoparticles within the standard limit of 1 mg per kg in a packaged food product, the fact that nanoparticles experience continual migration and release from the film over time substantiates the above assertion that some alternative antipathogenic materials and coatings reported to date achieve varied viability when potential human toxicity is considered. Questionable viability of Li et al.’s films as an antipathogenic contact killing surface also follows from the fact that the antibacterial agent, i.e., the biocidal nanoparticles, will migrate from the composite film over time, thus reducing antipathogenic performance over time too. Furthermore, the mechanical integrity of composite films also comes into question since the mechanical tensile strength and modulus of elasticity decreased with increased nanoparticle, i.e. increased antimicrobial agent, concentrations.

Though driven, at least partially, by the preemptive societal gains in health and wellness, the creatively exotic and unusual materials engineering solutions entertained within the global research community in an effort to tackle surface-contact-meditated transmission of infectious pathogens most likely follows from the conceivable fiscal reward that could be amassed. More clearly, such venturous research and development modalities being reported upon within the scholarly outlets of relevance was likely stimulated by monetary projections signifying that the marketplace for antimicrobial materials and surfaces will likely reach more than 8 billion USD by the mid-2020s. Given such a vast economic incentive, one more easily appreciates the rationale surrounding the reasons why such unorthodox coatings and surfaces were considered in the first place even most if not all of those listed above have not been recognized by relevant regulatory agencies as being dependably antimicrobial. As for material solutions that have been identified as consistently antipathogenic, the EPA offered researchers the scaffolding needed to reliably develop antimicrobial functional surfaces. As a result, nearly all of the EPA approved antimicrobial coatings and/or surfaces are required to contain greater than or equal to 60% copper content. Because of the fact that many of the alternative antipathogenic materials and coatings of significance did not utilize copper in accordance with the EPA, alternative antipathogenic copper-based surfaces are considered hereafter.

#### Copper-based alternative antimicrobial materials

Even though the range of materials, methods of fabrication, and materials processing/manufacturing techniques that may be entertained within this subsection of the present literature review are less wide ranging than that of the non-copper-based alternatives, numerable means of antimicrobial copper-containing materials and coatings production have been reported upon. Given the numerable production and fabrication approaches available for copper-based alternative antipathogenic copper-containing materials and coatings procurement, a handful of current research articles are situated within the array of antipathogenic copper surfaces published upon thus far. Hence, research by Haider et al. will be discussed first. Specifically, a hybrid poly-(lactide-co-glycolide) and copper-oxide nanoparticle containing composite nanofiber-based scaffolding was developed by Haider et al. by way of electrospinning [32]. The dependence upon the use of nanoparticles by Harider et al. raises concerns and questions surrounding the viability of extending Haider et al.’s electro-spun antimicrobial composite material to the realm of structurally sound contact killing surfaces in hospital and medical settings. Such concerns from the potential health effects and human toxicity associated with the ingestion of detached nanoparticles from the poly-(lactide-co-glycolide) base material. Nevertheless, Haider et al. noted that, at the very least, poly-(lactide-co-glycolide) on its own had been authorized for use by the U.S. Food and Drug Administration. In any case, antibacterial testing demonstrated inhibited bacterial growth of Gram-positive and Gram-negative strains, leading Hairder et al. to mechanistically attribute the antimicrobial efficacy of their scaffoldings to the presence of copper-oxide nanoparticles and Cu^2+^ ion diffusion from the copper-oxide housed within the composite material. While copper is known to be consistently oligodynamic, prior discussion by the present authors in the second section of this manuscript highlighted the fact that “cuprous oxide” is thought to be more bactericidal than “copper oxide” and Cu^1+^ ion diffusion is thought to be more bactericidal than Cu^2+^ ions as well. Therefore, Haider et al. would have likely achieved even greater antipathogenic performance if Cu_2_O nanoparticles were used in place of CuO nanoparticles.

From the vantage point of another composite based material, although comprised of a copper-zirconium-aluminum metallic glass composite rather than a poly-(lactide-co-glycolide) and metal-oxide nanoparticle composite in the case of Hairder et al., Villapun et al. published two studies centered upon the antimicrobial behavior of said copper-based metallic glass composites [33, 34]. In one of the studies, the antibacterial behavior of Villapun et al.’s copper-containing metallic glass composites of the stoichiometric form Cu_50 + x_(Zr_44_Al_6_)_50-x_, where x is either 0, 3 or 6. Villapun et al. noted that the crystallinity of the metallic glass composite increased proportionally with respect to the copper content. While Villapun et al. were also interested in the wear and mechanical properties of the composite compositions studied during the course of their research, the antimicrobial testing analysis identified the Cu_56_Zr_38.7_Al_5.3_ metallic glass composite composition as achieving greater antibacterial performance than the other forms after 1 h of exposure to Gram-negative *E. coli* and Gram-positive *Bacillus subtilis*. Each of the copper-based metallic glass composites were also found to completely eliminate the bacteria tested through a time-kill approach after no more than 250 min. After Villapun et al.’s initial 2017 article, Villapun et al.’s second article investigated similar properties after modifying the composites surface topographies prior to antimicrobial testing [33, 34].

Interestingly, Villapun et al. investigated the effects of grinding-induced surface roughness variation (from 240 grit to 4000 grit), as well as surface oxidation, upon the copper-based metallic glass composite’s *E. coli* contact killing efficacy. The authors asserted that variations in roughness were inconsequential in terms of the composites antipathogenic performance when *E. coli* (strain K12) bacteria was explored. Remarkably, the oxidized copper-based metallic glass composite procured by Villapun et al. increased the antimicrobial efficacy. Villapun et al.’s realization that Cu_2_O maintains greater bactericidal efficacy than that of CuO, which the authors of the present review also discussed above. Furthermore, the oxidized metallic glass composite entertained by Villapun et al. was found to have a multilayered oxidized micro/macro-structure near the surface too. In spite of the fact that the less antibacterial CuO was identified as the outer-most layer, the crystalline nature of the Cu_2_O-CuO layers was presented by Villapun et al. as the copper ion diffusion pathway framework required to understand the reason for the oxidized samples enhanced performance. Villapun et al.’s crystallinity proposal was consistent with work by Sousa et al. that detailed how polycrystalline copper’s grain boundaries act as diffusion highways for contact killing/inactivating copper ions [17].

Around the same time that Villapun et al. published their second work of scholarship, which was just discussed, Ciacotich et al. published an analysis of the antibacterial efficacy of an alloyed copper coating with silver as the alloying element [35]. To perform a proper investigation of the antipathogenic performance, the copper-silver coating was subjected to testing conditions according to an EPA protocol wherein a bacterial biofilm was imposed upon the surface of the alloyed copper-silver coating. Ciacotich et al. found that a Gram-positive bacterial biofilm was completely killed within 5 min of exposure time. Additional discussion surrounding their hypothesis that the bacterial contact killing associated with the copper-silver coating was a multifactorial and complexly intertwined process, dependent upon local variations in pH, copper ion diffusion and bacterial cell oxidation, among other mechanisms, was provided by the authors as well. Returning to commercially pure copper as the antimicrobial material, Kocaman et al. produced biocidal wire arc sprayed copper coatings using a twin wire arc spray gun and a stainless-steel substrate surface in [36]. Said otherwise, Kocaman et al. characterized the antibacterial efficacy of copper coatings using a wire arc spray deposition process after exposure to various bacterial pathogens. The pathogens explored consisted of MRSA, *P. aeruginosa,* Vancomycin-resistant Enterococcus (VRE), *E. coli*, and *S. aureus*.

Kocaman et al. found that *E. coli*, *S. aureus*, and *P. aeruginosa* “vanished from the surface” after 15 min of exposure time to the wire arc sprayed copper coating. Intriguingly, the Vancomycin-resistant Enterococcus (VRE) and MRSA superbugs required more time for complete contact killing and inactivation to occur. At 15 min, 100% of the MRSA was killed (initially) whereas only 96.11% of the VRE was passivated. At 1 h, 98% of the VRE was killed while 98.3% of the MRSA was killed. Finally, at 2 h, 99.7% of the VRE was killed while 100% of the MRSA was killed. The authors concluded their study by way of reiterating the fact that “no difference was observed in biocidal performance” between Gram-positive and Gram-negative bacteria. Kocaman et al. also provided a reasonable hypothesis as to why the superbugs required greater exposure times for complete contact killing based upon the fact that superbugs have been found to have “thicker cell membranes, possibly causing a decrease in the rate of ion diffusion through the cell wall.” It is the opinion of the authors of the present review article that the hypothesis provided by Kocaman et al. deserves continued investigation in future work. Once more, an additional copper-containing alternative may be noted herein concerns the work of Muralidharan, which was recounted upon in a master’s thesis from the University of Waterloo. Specifically, a sulphonated poly(ether-ether-ketone)-copper film for antipathogenic functionality was described by Muralidharan et al. in 2020 [37]. Just as Kocaman et al. is deserved of continued consideration, Muralidharan’s advancements deserve the same.

As for the materials science and biomechanical engineering community, a recent work of scholarship was released on a pre-print server, which documented the use of Luminore CopperTouch™ coatings to inactivate SARS-CoV-2 on coated surfaces [38]. During the course of Mantlo et al.’s research, Luminore CopperTouch™ copper surfaces were studied alongside a copper-nickel surface to investigate the inactivation of filoviruses as well as SARS-CoV-2. In doing so, Luminore CopperTouch™ copper as well as the copper-nickel surfaces were exposed to “viral titers in Vero cells from viral droplets” for at least 30 min and up to 2 h. As a result, the Luminore CopperTouch™ surface was found to inactivate 99% of the SARS-CoV-2 titers within 2 h. The Luminore CopperTouch™ copper surfaces were also found to inactivate 99.9% of the Ebolavirus and Marburgvirus in less than 2 h too. Unfortunately, the research by Mantlo et al. does not appear to delve into the realm of mechanisms associated with copper-mediated contact inactivation of SARS-CoV-2 [38].

Consistent with our own claim that cuprous oxide (Cu_2_O) is likely to be just as effective as pure copper in diffusing the atomic copper ions needed for viral contact inactivation [17], according to [39], recent work undertaken at Virginia Tech has identified another copper-based coating that can also rapidly inactivate SARS-CoV-2 [40]. Still, one of the most promising aspects of copper cold spray antipathogenic coatings relative to the coatings presented by Behzadinasab et al. and Mantlo et al. is the likelihood of even greater inactivation rates below 1-h of exposure time, given the dynamically recrystallized and severely plasticly deformed microstructure, which greatly enhances ion diffusivity of the copper surfaces via refined grains and therefore the significant portion of diffusive grain boundaries.

### Copper contact killing and inactivating mechanisms

There are many mechanisms at play that can lead to the inactivation of viruses and death of bacteria; however, this review will touch on those associated with contact killing on metallic surfaces. A study conducted by Kawakami et al. of Osaka City University, subjected Gram-positive *S. aureus* and Gram-negative *E. coli* bacteria to 21 elemental metals, of which copper and silver demonstrated 5-fold to 10-fold higher kill rates compared to the 19 other elements [41]. The iron on which these elements were deposited was shown to disrupt the membrane but was not indicative of being able to kill the 2 bacterial strains. The study concluded that while there was moderate toxicity involved with cobalt, nickel, and aluminum, the introduction of copper and silver had the most profound effects. Of course, the identification of Al as being moderately toxic to pathogens by Kawakami et al. raises questions surrounding the method of analysis that the researchers employed.

**Fig. 6:**
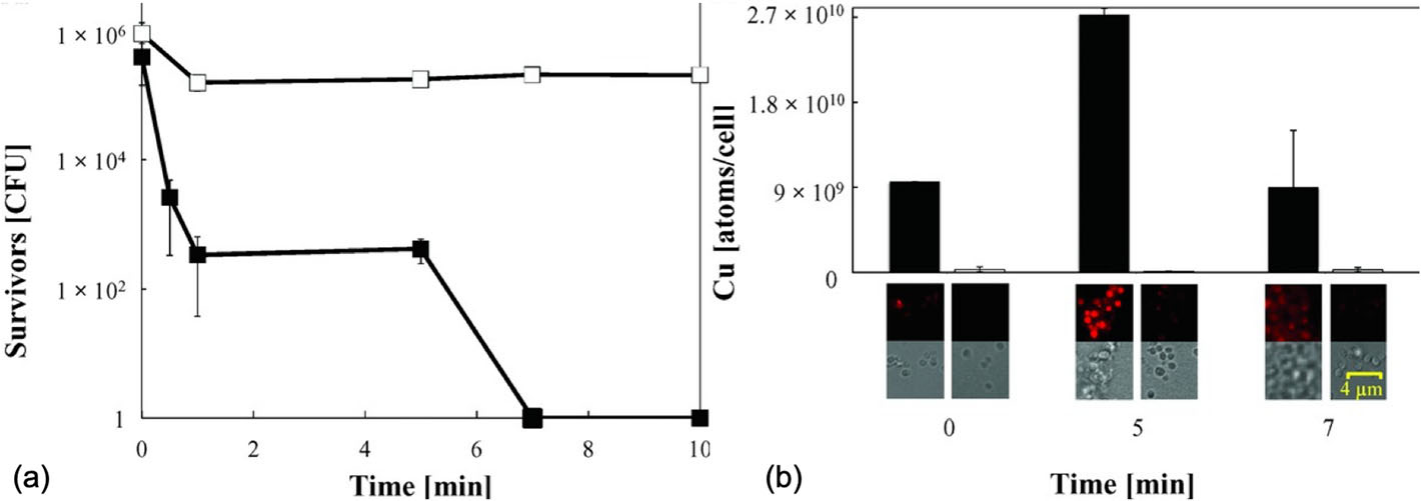
Taken from Santo et al. to demonstrate “Staphylococcus haemolyticus is rapidly killed on dry metallic copper (Cu) surfaces and cells accumulate large amounts of Cu. Cells of S. haemolyticus were exposed to dry metallic Cu surfaces or stainless steel for the indicated times, removed, washed, and plated on solidified growth media”

Nevertheless, it has also been regarded that in copper and iron, the oxidized ion couples share similar redox potentials and can catalyze Fenton chemistry. This is based on the reactive oxygen species (ROS) which is exceptionally volatile to lactic acid bacteria as they produce hydrogen peroxide which can cause irreparable damage to cellular components when exposed to copper ions. Moreover, pertaining specifically to copper ions, the cytotoxin killing mechanism has been hypothesized as occurring as the cells uptake massive amounts of copper, which in *E. coli*, for example, would displace 4Fe-S4 clusters, therefore resulting in dehydratases. However, Fenton chemistry related phenomena are not universally accepted as the mechanism most responsible for contact killing [42]. Membrane damage was evident in copper exposed Gram-negative bacteria, *E. coli*, as proteomic profiling elicited that copper had upregulated cell envelope and capsule polysaccharide biogenesis proteins. In viruses, while the structures vary significantly, some of the same kill mechanisms hold as copper ions overflow the cells and can cause extensive damage to the membrane through oxidative damage resulting in fully compromised cell structure [43]. Figure 6 is adopted from Santo et al. as a result.

#### Copper cold Spray’s microstructurally driven Antipathogenic 794 performance

Having already introduced one of many proposed mechanisms associated with copper-mediated contact killing/inactivation through atomic ion inactivation with a pathogen in intimate contact with a given bio-functional copper surface, let us now turn our attention to the matter of antipathogenic behavior of copper cold spray coatings. In most of the pathogen-copper killing/inactivation interactions reported upon to date, such as genomic damage, membrane disruption and damage, ROS, or atomic copper ion speciation [44], microstructural and physical pathways for copper ion diffusion to the infectious agent from the copper material must be achieved. As briefly discussed earlier, Champagne et al. attributed the unique antipathogenic performance of copper cold spray coatings to the “extreme work hardening and correspondingly high dislocation density within the deposit… and ionic diffusion occurs principally through these dislocations…” associated with the supersonic particle consolidation process [6].

**Fig. 7:**
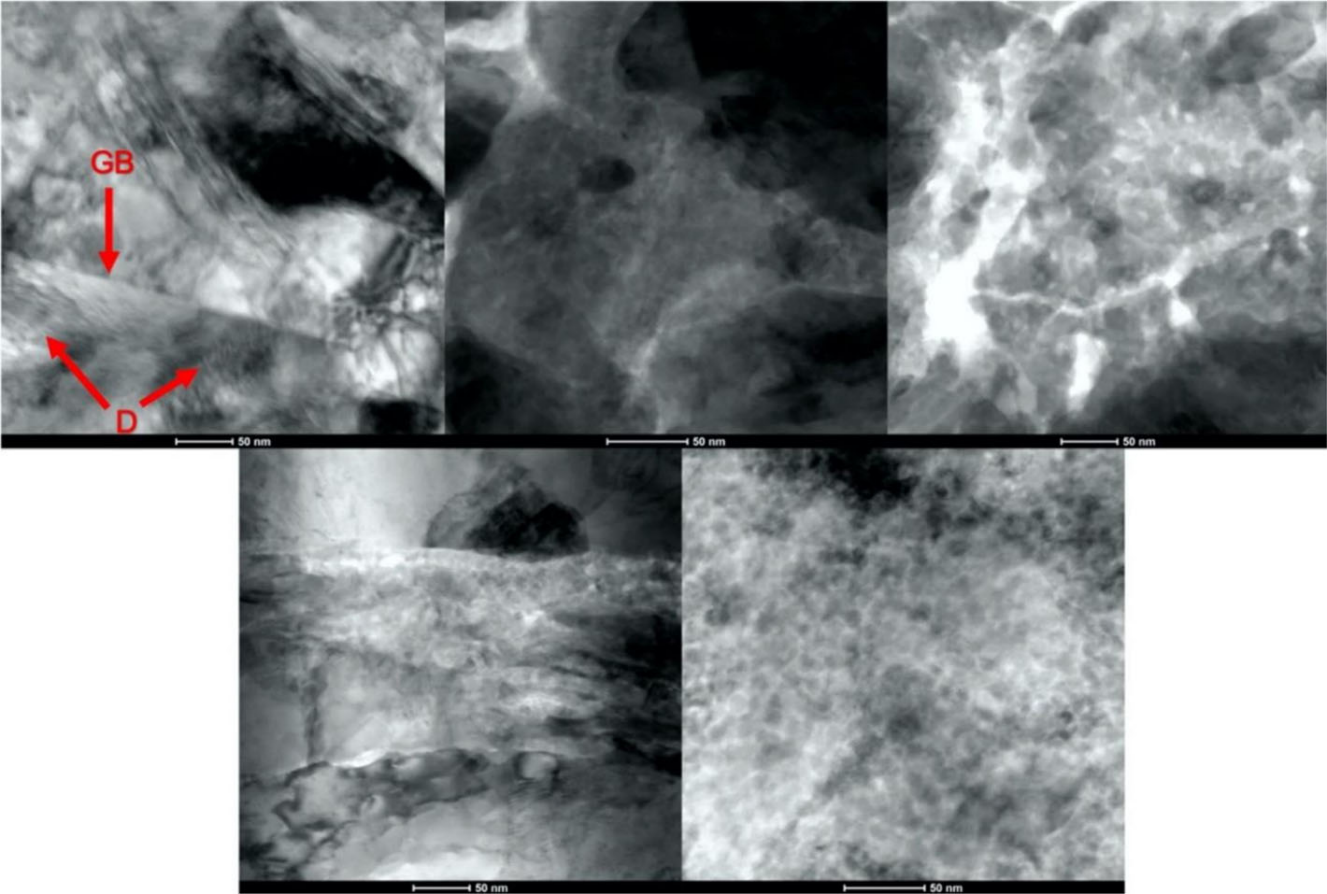
Unique microstructure associated with nanostructured copper cold spray coatings studied by Sousa et al. [17]. Note that the embedded use of “GB” refers to grain boundaries and the embedded us of “D” refers to dislocations or dislocation structures

Succeeding articles were published by Champagne et al., among others who collaborated with Champagne, in an effort to support and substantiate Champagne et al.’s dislocation-driven hypothesis. This was pursued by way of invoking a mechanical relationship between dislocation density and hardness, wherein greater hardness’s were treated as a marker of greater antipathogenic functioning for copper cold spray material consolidations [45]. However, Sousa et al. began to further analyze the microstructures and mechanical behavior of the antimicrobial copper cold spray coatings to probe the appropriateness of Champagne et al.’s dislocation density driven atomic copper ion diffusion framework for evaluating the improved contact killing and/or inactivation efficacies for both viral and microbial agents in comparison with alternative bactericidal and viricidal materials [46]. As a result, the most current assessment and research by Sousa et al. offers a comprehensive examination of the role dislocations retain as compared to the role of grain-boundary mediated atomic copper ion diffusion [17]. Figure 7 captures the unique microstructure associated with nanostructured copper cold spray coatings studied by Sousa et al.

Fittingly, numerable studies have also arisen that validate superior atomic mobility and diffusion via grain boundaries in comparison with dislocations, signifying that the grain boundary-mediated pathway should to be considered by those pursuing the enactment of copper cold spray surfaces as a preemptive measure against fomite transmission of pathogens. With the aforementioned in mind, the declaration that emphasis and optimization must to be given to grain boundaries housed in antimicrobial copper cold spray material consolidations does not mean that the role dislocations can play should be disregard. By taking a mutualistic approach that preserves the maximal surface area concentration of greatly disordered grain boundaries whilst also retaining an increased density of dislocations within a copper cold spray surface, a heightened rate of atomic copper ion diffusion may be achieved. However, such a synergistic approach ought to not be pursued at the cost of forfeiting the grain boundary concentration nor should such a compounded effect be pursued if the dislocations take on a disadvantageous form and atomic copper ion diffusion sink. Nonetheless, some work has been done attesting to the fact that dislocations may generally improve diffusion irrespective of being a screw or an edge dislocation type, for example.

In fact, research in support of dislocation-driven and line-defect-dominated atomic copper ion diffusion as the microstructural feature liable for enhanced antipathogenic performance has been reported upon [47]. Unfortunately, said work’s hyper focus upon dislocations overshadowed any nuanced and explicit contemplation surrounding grain size as a potential driving force for atomic copper ion diffusion. If explicit consideration was assigned to grain size within the copper system studied [47], their work would have also potentially attested to the area-fraction-containing grain boundaries as being integral to increased antipathogenic activity of copper, due to the fact that X-ray diffraction derived crystallite sizes were found to decrease concurrently with the increased dislocation density.

### Copper cold spray in light of SARS-CoV-2

Given the current climate surrounding the ongoing COVID-19 global pandemic (at the time of penning the present review article), which is caused by the novel coronavirus known as SARS-CoV-2, continued discussion on antiviral copper-containing alternatives will be discussed herein. As of 16 January 2021, the WHO’s total global confirmed case count around the world indicated that more than 92 million COVID-19 cases have been recorded alongside at least 2 million confirmed deaths from COVID-19 too [48]. With such a human toll, as well as the continued spread of the virus, the international medical, engineering, and scientific communities have coalesced around the pandemic by way of dedicating resources, research, and development, in an effort to prevent as well as combat further SARS-CoV-2 transmission.

**Fig. 8:**
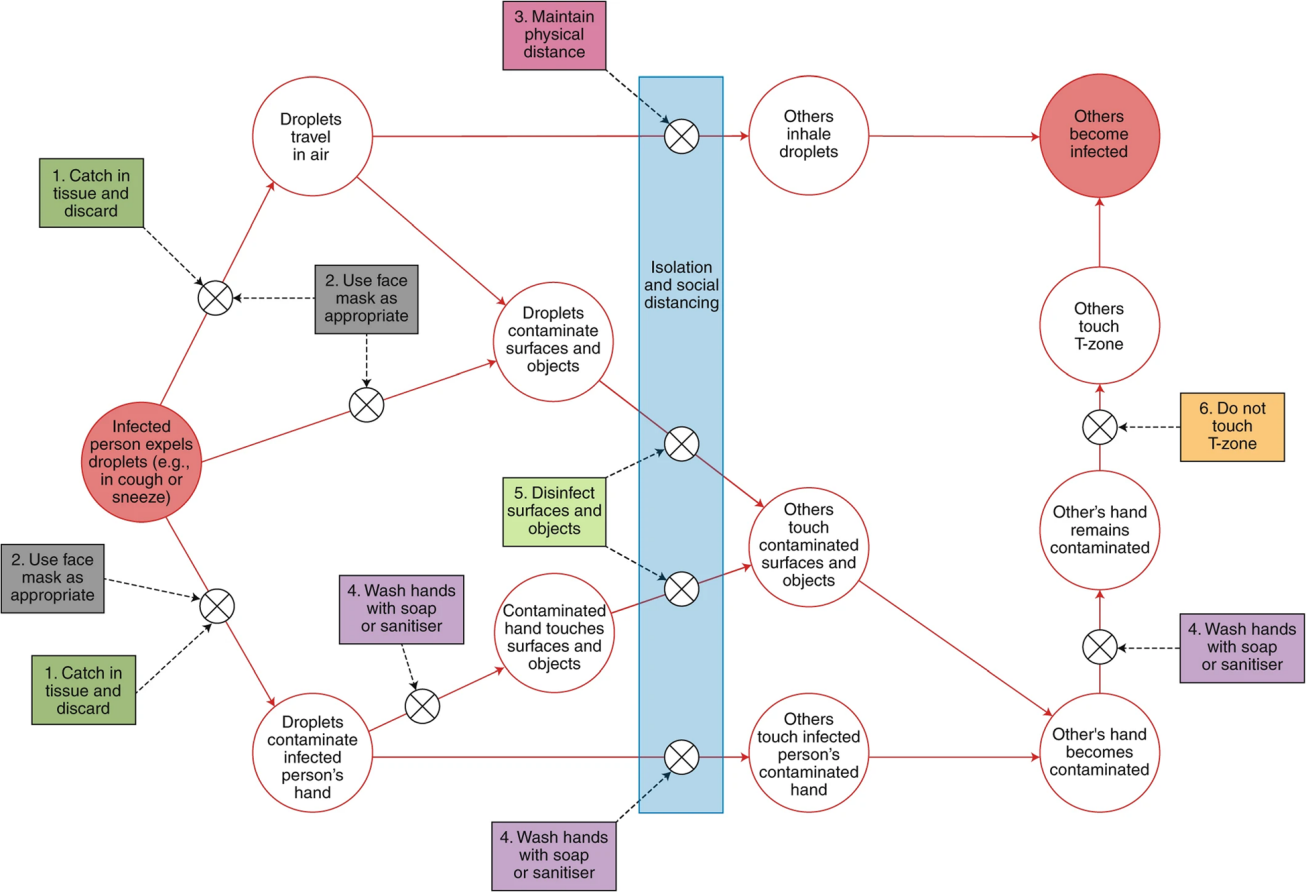
“Transmission pathways for SARS-CoV-2 and behaviors needed to block these. The arrows to the left of the blue bar relate to the infected person while those to the right relate to other people who may become infected,” [50, 51].

As discussed within a previous subsection of the present review article, influenza A virions were found to achieve successful inactivation after 2 h (or fewer) of intimate and direct exposure to commercially pure copper cold spray coatings reported upon by Sundberg et al. [8, 15, 18, 45] Champagne et al. [16] and Sousa et al. [17], one may assuredly hypothesize that copper cold spray surfaces would also successfully inactivate SARS-CoV-2 too. Such an informed hypothesis not only stems from the fact that copper cold spray antimicrobial consolidations have been identified as an anti-influenza A bio-functional surface; rather, it also invokes related findings recently reported upon that SARS-CoV-2 was able to be completely inactivated on a less antiviral copper surface than that of the cold sprayed copper coatings [49]. Keeping with this line of discourse, it stands to reason that anti-SARS-CoV-2 copper cold spray coatings could be rapidly deployed as a preventative measure in so far as COVID-19 is concerned. Beyond the benefits achieved through quick introduction of copper cold spray surfaces to the suitable high-touch infrastructure in so far as SARS-CoV-2 is concerned, continued security and the mitigation of forthcoming pandemics through the prevention of bacterial as well as non-SARS-CoV-2 fomite transmission. Fig. 8 illustrates the prospective fomite transmission pathways associated with SARS-CoV-2 according to [50, 51].

Said hypothesis is consistent with statements released by the CDC which recognized that “it may be possible that a person can get COVID-19 by touching a surface or object that has the virus [SARS-CoV-2] on it and then touching their own mouth, nose, or possibly their eyes” according to an interpretation distributed by the CDC in May 2020 [52]. Prior to the public release of the CDC clarification just mentioned, Han et al. corroborated the necessity for pathogen inactivating surfaces in clinical, medical, and environments that house a notable concentration of high-touch surfaces, as a mitigative measure in the against SARS-CoV-2 [53]. Following the findings reported by Han et al. and the public perspective issued by the CDC, the World Health Organization (WHO) released a scientific briefing that attested to the veracity associated with contact-mediated transmission of the SARS-CoV-2 virus responsible for the ongoing COVID-19 public health crisis and global pandemic [54].

The environments that house a notable concentration of high-touch surfaces include nursing homes, medical facilities, active public transportation, and schools, and have developed into focal points for the spread and transmission of the SARS-CoV-2 virus during the current COVID-19 pandemic. By way of refurbishing said surfaces within environments that house the greatest concentration of fomite transmission focal points in the most vulnerable and hard-hit geographies with such antiviral copper cold spray coatings, the functionalized material consolidations would contribute to the alleviation and inhibition of SARS-CoV-2 infection. Distinctive copper coating manufacturing processes accomplish variable sterilization rates, even when feedstock materials of the same composition are consumed during antimicrobial surface generation. Copper coatings deposited on a traditional hospital-grade surface via cold spray kill bacteria and inactivate viral pathogens with commendable speed. As a controllable and versatile coating technology, cold spray is especially appropriate for covering hospital equipment and vulnerable touch surfaces found in clinical settings. In fact, the materials science community has also started to advocate for the antipathogenic functionalization of common touch surfaces in public areas through the use of copper in the fight against COVID-19 too [55–59].

## Data Availability

Not applicable.

## Abbreviations

COVID-19: Coronavirus Disease 2019
ROS: Reactive Oxygen Species
4Fe-S4: Iron-Sulfur Protein
Cu: Copper
WHO: World Health Organization
ARL: Army Research Laboratory
WPI: Worcester Polytechnic Institute
LLC: Limited Liability Corporation
PSU: Penn State University
UMass: University Of Massachusetts
MRSA: Methicillin-Resistant *Staphylococcus aureus*
EPA: Environmental Protection Agency
*D*_*eff*_: Effective Ionic Diffusivity
*D*_*o*_: Lattice Diffusivity
*a*: Average Dislocation Radius
*D*_*d*_: Pipe Dislocation Diffusivity
*ρ*_*d*_: Dislocation Density
*ρ*: Dislocation Density
*f*_*i*_: Fraction Of Atoms Undergoing Atomic Diffusion Via The & Additional Interfacial Pathways Within A Material
*D*_*i*_: Diffusion Coefficient For Atomic Diffusion Through Additional Interfaces Within A Material
*δ*_*gb*_: Grain Boundary Thickness
*w*: Line Width
*d*: Average Grain Or Sub-Grain Size
*D*_*gb*_: Diffusion Coefficient For Atomic Diffusion Through The Grain Boundaries
*D*_*C*_: Diffusion Coefficient For Atomic Diffusion Through Dislocation Cores
ConvCu: Conventional Copper Coating
NanoCu: Nanostructured Copper Coating
AFM: Atomic Force Microscopy
ASME: American Society of Mechanical Engineers
ISO: International Standards Organization
PEEK: Polyether Ether Ketone
U.S.: United States
USD: U.S. Dollar
HIV: Human Immunodeficiency Viruses
AIDS: Acquired Immunodeficiency Syndrome
CDC: Centers for Disease Control and Prevention
SARS-CoV-2: Severe Acute Respiratory Syndrome Coronavirus 2
VRE: Vancomycin-resistant Enterococci
